# The fructose tolerance test in patients with chronic kidney disease and metabolic syndrome in comparison to healthy controls

**DOI:** 10.1186/s12882-015-0048-y

**Published:** 2015-05-03

**Authors:** Rafał Donderski, Ilona Miśkowiec-Wiśniewska, Marek Kretowicz, Magdalena Grajewska, Jacek Manitius, Anna Kamińska, Roman Junik, Joanna Siódmiak, Anna Stefańska, Grażyna Odrowąż-Sypniewska, Agnieszka Pluta, Miguel Lanaspa, Richard J Johnson

**Affiliations:** Department of Nephrology, Hypertension and Internal Medicine, Collegium Medicum, Bydgoszcz, Nicolaus Copernicus University, Toruń, Poland; Department of Diabetology and Endocrinology, Collegium Medicum, Bydgoszcz, Nicolaus Copernicus University, Toruń, Poland; Department of Laboratory Medicine, Collegium Medicum, Bydgoszcz, Nicolaus Copernicus University, Toruń, Poland; Institute of Public Nursing, Collegium Medicum, Bydgoszcz, Nicolaus Copernicus University, Toruń, Poland; Division of Renal Diseases and Hypertension, University of Colorado Denver, Denver, CO USA

**Keywords:** Chronic kidney disease, Obesity and metabolic syndrome, Fructose tolerance test, Uric acid, Organ damage

## Abstract

**Background:**

Fructose acutely raises serum uric acid in normal subjects, but the effect in subjects with metabolic syndrome or subjects with chronic kidney disease is unknown. The aim of the study was to evaluate changes in serum uric acid during the fructose tolerance test in patients with chronic kidney disease, metabolic syndrome with comparison to healthy controls.

**Methods:**

Studies were performed in 36 subjects with obesity (body mass index >30) and metabolic syndrome, 14 patients with stage 3 chronic kidney disease, and 25 healthy volunteers. The fructose tolerance test was performed in each patient. The change in serum uric acid during the fructose challenge was correlated with baseline ambulatory blood pressure, serum uric acid, metabolic, and inflammatory markers, and target organ injury including carotid intima media thickness and renal resistive index (determined by Doppler).

**Results:**

Absolute serum uric acid values were highest in the chronic kidney disease group, followed by the metabolic syndrome and then healthy controls. Similar increases in serum uric acid in response to the fructose tolerance test was observed in all three groups, but the greatest percent rise was observed in healthy controls compared to the other two groups. No significant association was shown between the relative rise in uric acid and clinical or inflammatory parameters associated with kidney disease (albuminuria, eGFR) or metabolic syndrome.

**Conclusions:**

Subjects with chronic kidney disease and metabolic syndrome have higher absolute uric acid values following a fructose tolerance test, but show a relatively smaller percent increase in serum *uric acid*. Changes in serum uric acid during the fructose tolerance test did not correlate with changes in metabolic parameters, inflammatory mediators or with target organ injury. These studies suggest that acute changes in serum uric acid in response to fructose do not predict the metabolic phenotype or presence of inflammatory mediators in subjects with obesity, metabolic syndrome or chronic kidney disease.

**Trial registration:**

The study was registered in ClinicalTrials.gov. Identifier : NCT01332526. www.register.clinicaltrials.gov/01332526

**Electronic supplementary material:**

The online version of this article (doi:10.1186/s12882-015-0048-y) contains supplementary material, which is available to authorized users.

## Background

Excesive consumption of added sugars such as high-fructose corn syrup (HFCS) increases the risk for cardiovascular diseases, the metabolic syndrome, type 2 diabetes, dyslipidemia, fatty liver and chronic kidney disease (CKD) [1-5]. Fructose, a major component of added sugars, is relatively unique among carbohydrates in its ability to increase uric acid generation, which occurs during the metabolism of fructose. The rise in serum uric acid has been proposed to be one mechanism by which fructose may increase the risk for hypertension and CKD progression [6-8]. Fructose feeding with increased serum uric acid has been shown in rats to cause an afferent arteriolopathy, glomerular hypertension, glomerulosclerosis and tubulointerstitial fibrosis [9-12].

The ability of fructose to raise serum uric acid has led to the development of a fructose tolerance test (FTT), in which an oral load of fructose is followed by measurement of serum uric acid over one to two hours. There is some evidence that the FTT is enhanced in subjects on a high fructose diet, as well as in subjects with hypertension [13]. Less is known about the changes of serum uric acid that occur during oral FTT in subjects with CKD and/or the metabolic syndrome. Anderstam analyzed the effect of 75 g of fructose load on serum uric acid in hemodialysis patients in comparison to healthy controls. They reported stable serum uric acid levels during the test in healthy individuals but a significant increase by 10% in hemodialysis patients at 240 minutes after fructose intake [14]. Here we compared the effects of FTT in subjects with obesity and metabolic syndrome, subjects with CKD and healthy controls. We also tested the hypothesis that the change in serum uric acid in response to fructose might correlated with the metabolic phenotype, the degree of renal injury, the presence of inflammation or the presence of target organ injury.

## Methods

Thirty-six patients with body mass index (BMI) >30 and metabolic syndrome (according to NCEP ATP III criteria) (mean age 53 ± 8; F-19,M-17); 14 patients with CKD stage 3 (mean age 67 ± 10; F-9, M-5) and 25 healthy volunteers (mean age 53 ± 10; F-15, M-10) were studied. Causes of CKD were: hypertensive nephropathy in 8 subjects, ischemic nephropathy in 3, chronic glomerulonephritis in 2, and chronic tubulointerstitial nephritis in 1.

### Inclusion criteria

Age 18–78 years, BMI >30 with metabolic syndrome (NCEP ATP III), CKD stage 3 with proteinuria less than 3.5 g per day, with a blood pressure <140/90 mmHg/.

### Exclusion criteria

Fructose intolerance, BMI < 18, presence of diabetes mellitus, symptomatic gout defined as an acute inflammatory arthritis in the last 3 months, or presence of chronic inflammatory diseases (including systemic lupus erythematosus - SLE, chronic inflammatory bowel disease, and rheumatoid arthritis) or use o immunosuppressive treatment, angiotensin converting enzyme (ACE) inhibitors, angiotensin receptor blockers (ARBs), or allopurinol.

### Laboratory analyses

Morning blood samples were evaluated after an overnight fast for creatinine, uric acid, sodium, potassium, glucose, fructose, insulin (insulin ELISA), triglycerides, HDL, LDL cholesterol, calcium, phosphorus, N-acetyl-beta-(D)-glucosaminidase (NAG) was assayed by colorimetric method (Roche Diagnostics), high sensitvity C reactive protein (hsCRP) was measured using the BN II System nephelometer (High Sensitivity CRP; Siemens Healthcare Diagnostics, IL, USA), monocyte chemoattractant protein-1 (MCP-1); was assayed using Human ELISA Kit, erythropoietin (EPO) was assayed using Human Erythropoetin ELISA Kit) tissue necrosis factor-alpha (TNF-alpha) was assayed using TNF-alpha Human ELISA Kit, tissue growth factor-beta (TGF-beta) was assayed using TGF-beta Human ELISA Kit, inducible nitric oxide synthase (iNOS) was assayed using Human inducible nitric oxide synthase, endothelial human nitric synthase (eNOS) was assayed using Human Endothelial nitric oxide synthase ELISA kit, endothelin-1 was assayed using Human Endothelin 1 ELISA Kit and cystatin C was assayed using human Cystatin C Quantikine ELISA Kit.

The same day a fructose tolerance test (FTT) was conducted in all investigated patients and controls. The standard FTT was performed after overnight fasting according to the same protocol. After giving 1 gram/kg body weight of fructose orally, blood was collected at 0, 30, 60 and 120 min for serum uric acid. The uric acid area under the curve was calculated. The day before blood sampling and the FFT test, a 24-hr urine collection for: sodium, calcium, phosphorus, creatinine, uric acid, and albumin was performed. Before the urine collection was initiated, ambulatory blood pressure (ABPM) was initiated, and anthropometric measurements including BMI and waist circumference were performed. Target organ damage was also assessed, including carotid ultrasound to measure intima medial thickness (IMT), renal artery Doppler to measure renal resistive index (RI), and severity of renal injury (albuminuria, eGFR). These measurements were performed the day before the FTT.

The study protocol was approved by the local Bioethical Committee of Collegium Medicum in Bydgoszcz, the Nicolaus Copernicus University in Toruń in 2011 [KB 91/2011]. Written informed consent was obtained from all participants of the study.

### Statistical analysis

Results are expressed as mean ± SD. Data were compared using Mann–Whitney non-parametrical tests. For all tests p value <0.05 was considered as statistically significant.

## Results

Laboratory data and anthropometric measurements in subjects with metabolic syndrome (BMI > 30), CKD and control groups are presented in Table 1 and Additional file 1: Table S1. As expected, subjects with CKD had lower eGFR and more microalbuminuria than healthy controls, and subjects with metabolic syndromes had worse lipid profiles and higher BMI compared to healthy subjects. Levels of inflammatory mediators and target organ injury were also greater in the subjects with metabolic syndrome and CKD compare to healthy controls (Additional file 1: Table S1). Carotid intimal medial thickness (IMT) was highest in CKD patients which may indicate advanced vascular lesions in that population. Blood pressure measurements in various study groups were presented in Table 2.

**Table 1:** **Laboratory and anthropometric measurements data of investigated groups: BMI > 30 (n = 36), CKD (n = 14) and control (n =25)**

	**BMI > 30** ^**(1)**^	**CKD** ^**(2)**^	**Control** ^**(3)**^	**p**
	**N = 36**	**N = 14**	**N = 25**	
	**Mean ± SD**	**Mean ± SD**	**Mean ± SD**	
	**Median (range)**	**Median (range)**	**Median (range)**	
Age [years]	52,6 ± 8,4	66,8 ± 9,9	53,2 ± 10,5	1-2 p = 0,0009
				1-3 p = 0,9672
				2-3 p = 0,0015
Weight [kg]	102,5 ± 14,8	79,2 ± 15,3	68,1 ± 10,6	1-2 p = 0,0003
				1-3 p = 0,0001
				2-3 p = 0,1200
BMI [kg/m^2^]	34,9 ± 4,5	27,7 ± 4,4	23,9 ± 2,8	1-2 p = 0,0002
				1-3 p = 0,0001
				2-3 p = 0,0598
Waist circumference [cm]	113,1 ± 11,6	100,5 ± 13,5	81,4 ± 9,6	1-2 p = 0,0168
				1-3 p = 0,0001
				2-3 p = 0,0003
Serum fasting glucose [mg/dl]	99,5 ± 14,2	100,6 ± 24,0	83,5 ± 8,4	1-2 p = 0,9807
				1-3 p = 0,0011
				2-3 p = 0,0103
Total cholesterol[mg/dl]	195,1 ± 40,5	198,4 ± 35,1	210,4 ± 48,9	p = ns
HDL [mg/dl]	47,5 ± 13,4	49,6 ± 14,9	59,8 ± 19,6	1-2 p = 0,9360
				1-3 p = 0,0225
				2-3 p = 0,2184
TG [mg/dl]	126 (57 – 1242)	119 (61 – 1242)	96 (54 – 142)	1-2 p = 1,0000
				1-3 p = 0,0040
				2-3 p = 0,1264
Serum uric acid [mg/dl]	5,72 ± 1,11	7,26 ± 2,21	4,52 ± 0,96	1-2 p = 0,0092
				1-3 p = 0,0059
				2-3 p = 0,0001
CRP [ng/l]	2,36 (0,27-106)	2,43 (0,20-41,9)	0,70 (0,11-17,81)	1-2 p = 1,0000
				1-3 p = 0,0016
				2-3 p = 0,0167
eGFR (MDRD equation) [ml/min]	95,1 ± 15,2	52,4 ± 21,0	98,5 ± 19,6	1-2 p = 0,0001
				1-3 p = 0,7730
				2-3 p = 0,0001
Serum creatinine[mg/dl]	0,81 ± 0,19	1,35 ± 0,38	0,78 ± 0,18	1-2 p = 0,0001
				1-3 p = 0,9230
				2-3 p = 0,0001
Urine uric acid mg/24 h	665 ± 350	340 ± 121	535 ± 196	1-2 p = 0,0070
				1-3 p = 0,2217
				2-3 p = 0,1507
Urine sodium mmol/l	135,9 ± 52,3	87,0 ± 39,9	96,6 ± 44,1	1-2 p = 0,0222
				1-3 p = 0,0129
				2-3 p = 0,8552
Urine sodium g/24 h	5,50 ± 2,22	4,15 ± 2,29	3,87 ± 1,52	1-2 p = 0,1865
				1-3 p = 0,0157
				2-3 p = 0,9310
Microalbuminuria/g creatinine	4,8 (1,3-830)	41,3 (3,7-2656)	4,2 (2,3-10,1)	1-2 p = 0,0146
				1-3 p = 1,0000
				2-3 p = 0,0021

Abbreviations and explanations: BMI – body mass index, HDL-high density cholesterol, TG –triglicerides, CRP- C reacting protein, NAG - N-acetyl-β-(D)-glucosaminidase, TNFα -tissue necrosis factor-α, TGFβ- tissue growth factor-β, iNOS- inducible nitric oxide synthase, eNOS- endothelial nitric oxide synthase, p < 0,05 –statistically significant.

**Table 2:** **ABPM measurements in various study groups: BMI > 30 (n = 36), CKD (n = 14) and control (n = 25)**

	**BMI > 30** ^**(1)**^	**CKD** ^**(2)**^	**Control** ^**(3)**^	**p**
	**N = 36**	**N = 14**	**N = 25**	
	**Mean ± SD**	**Mean ± SD**	**Mean ± SD**	
	**Median (range)**	**Median (range)**	**Median (range)**	
SBP[ABPM -24 h]	128,5 ± 12,1	138,8 ± 22,9	122,1 ± 9,5	1-2 p = 0,1345
				1-3 p = 0,2416
				2-3 p = 0,0066
DBP [ABPM-24 h]	76,3 ± 9,4	79,9 ± 6,8	73,5 ± 4,4	p = ns

Abbreviations: SBP –systolic blood pressure, DBP-diastolic blood pressure, ABPM –ambulatory blood pressure monitoring.

(1) means - BMI> 30 group; (2) means - CKD group; (3) means - control group.

### Uric acid

**Figure 1:**
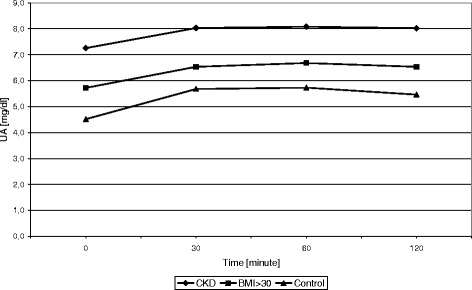
Changes in URIC ACID concentration during FTT in BMI>30; CKD, and control group.

Serum uric acid increased during the FTT in all three groups, peaking at approximately 60 minutes (Figure 1). At each time point, serum uric acid was higher in the subjects with metabolic syndrome and the subjects with CKD compared to healthy controls (Table 3). Nevertheless, the absolute increase in serum uric acid was not different among groups, although it tended to be highest in the healthy controls. This was reflected by a greater percent change in serum uric acid in the healthy controls compared to the CKD and metabolic syndrome groups (Table 3).

**Table 3:** **Comparison of investigated groups following administration of fructose (FTT)**

	**BMI > 30** ^**(1)**^	**CKD** ^**(2)**^	**Control** ^**(3)**^	**p**
	**Mean ± SD**	**Mean ± SD**	**Mean ± SD**	
URIC ACID (FTT) 0	5,72 ± 1,11	7,26 ± 2,21	4,52 ± 0,96	1-2 p = 0,0092
				1-3 p = 0,0059
				2-3 p = 0,0001
URIC ACID (FTT) +30 min	6,53 ± 1,30	8,03 ± 2,24	5,68 ± 1,15	1-2 p = 0,0240
				1-3 p = 0,1076
				2-3 p = 0,0003
URIC ACID (FTT) +60 min	6,68 ± 1,29	8,08 ± 2,19	5,73 ± 1,02	1-2 p = 0,0300
				1-3 p = 0,0542
				2-3 p = 0,0002
URIC ACID (FTT) +120 min	6,53 ± 1,22	8,02 ± 2,27	5,46 ± 1,05	1-2 p = 0,0196
				1-3 p = 0,0261
				2-3 p = 0,0001
AUC for URIC ACID	778 ± 148	954 ± 266	660 ± 123	1-2 p = 0,0203
				1-3 p = 0,0403
				2-3 p = 0,0002
% Δ (URIC ACID60 vs URIC ACID 0)	17,0 ± 8,9	12,5 ± 10,1	28,3 ± 14,5	1-2 p = 0,5552
				1-3 p = 0,0020
				2-3 p = 0,0013

(1) means - BMI>30 group; (2) means CKD group; (3) means - control group.

We had hypothesized that the degree of rise in serum uric acid might correlate with the baseline phenotype of the individual. However, there was no relationship between changes in uric acid with any of the baseline clinical parameters in our study, including eGFR, microalbuminuria, or markers of metabolic syndrome (systolic and diastolic blood pressure, serum triglycerides, serum HDL cholesterol, fasting blood glucose, or BMI), inflammation or target organ injury (see Table 4).

**Table 4:** **Linear correlation for** Δ**URIC ACID 60- URIC ACID 0 in FTT with selected parameters**

**Parametr**	**CKD**		**Metabolic syndrome**		**Control group**	
	**Δ URIC ACID60-URIC ACID0**		**Δ URIC ACID60-URIC ACID0**		**Δ URIC ACID60-URIC ACID0**	
Weight [cm]	−0,48	p=,086	−0,31	p=,067	−0,32	p=,125
BMI [kg/m^2^]	−0,39	p=,172	−0,36	p=,032	−0,21	p=,326
Triglicerydes [mmo/l]	−0,45	p=,103	−0,35	p=,033	−0,26	p=,208
eGFR [ml/min]	0,12	p=,676	−0,01	p=,969	−0,21	p=,308
IMT [mm]	0,04	p=,902	−0,17	p=,341	−0,05	p=,811
RI	−0,07	p=,835	0,34	p=,052	0,07	p=,747
PI	−0,03	p=,927	0,34	p=,051	−0,07	p=,761

Abbreviations: IMT- intima media thickness, RI-resistance index PI- pulsatility index. p < 0,05 –statistically significant.

## Discussion

The ability of fructose to increase serum uric acid was discovered in the 1960s, and could be quantified by a “fructose tolerance test” in which an oral dose of fructose is followed by serial measurements of serum uric acid [15-21]. Subsequent studies showed that the increase in serum uric acid was increased in subjects receiving a high fructose diet, and could be reduced by dietary fructose restriction [13]. Here we examined the effect of fructose on the uric acid response of subjects with obesity and metabolic syndrome, subjects with chronic kidney disease, and healthy controls. Our hypothesis was that subjects with CKD or obesity/metabolic syndrome might show a relatively greater increase in serum uric acid compared to healthy controls. This is because there is some evidence that subjects who are overweight or insulin resistant are more sensitive to the metabolic effects of fructose [22,23]. In addition, uric acid is known to regulate fructokinase [24], the principal enzyme driving fructose metabolism, and hence we expected a greater metabolic response to fructose in subjects with CKD or metabolic syndrome in which baseline uric acid levels tend to be higher than in healthy controls.

The principal findings, however, could not confirm the hypothesis. We did see higher baseline uric acid levels in both subjects with obesity/metabolic syndrome or CKD, and following an oral fructose load the uric acid levels rose further, peaking at 30 to 60 minutes (Figure 1). Indeed, absolute values of uric acid were always higher in these two groups of subjects compared to healthy controls. However, there was no difference in the absolute increase in uric acid level between the three groups. Furthermore, the healthy controls actually showed a greater relative percent increase in uric acid compared to the two other groups.

The significance of these findings remain uncertain. The relatively less uric acid response in subjects with CKD and/or metabolic syndrome might reflect blunted xanthine oxidase activity, since uric acid can cause a feedback inhibition of xanthine oxidase[25]. It is also possible that there could be an inhibition of AMP deaminase, an upstream enzyme activated by fructose. AMP deaminase is suppressed by phosphate, and serum phosphate levels tend to be high in subjects with CKD [26]. Furthermore, there is the possibility that fatty liver itself, which is common in subjects with high uric acid levels, including those with CKD or metabolic syndrome, might also have feedback to block uric acid generation in response to fructose [27].

It is also possible that the relatively blunted rise in serum uric acid could reflect differences in urinary uric acid responses to the fructose. Baseline studies did show that the subjects with metabolic syndrome had higher urinary sodium levels, and high urinary sodium can increase uric acid excretion. However, we administered the fructose tolerance test after an overnight fast and no subjects received salt during this time. However, we do recognize that a limitation of our study is that we did not measure urinary uric acid excretion during the fructose tolerance test, so it is possible this could explain the relatively blunted rise in uric acid in our subjects with metabolic syndrome. Likewise, it is also known that subjects with CKD show increased gut excretion of uric acid, and a compensatory mechanism could also be operative in these subjects.

We also tested the hypothesis that the degree of change in uric acid with the fructose tolerance test might correlate with metabolic, inflammatory, renal or target organ injury in these subjects. However, this was not observed. We conclude that the fructose tolerance test is unlikely to be able to be used as a way to predict the metabolic phenotype of the patient.

While these studies suggest a limited usefulness of the fructose tolerance test, the potential importance of fructose as a mediator of kidney disease or metabolic syndrome should not be dismissed based on the current study. Indeed, recently our group evaluated the effect of dietary fructose restriction for 6 weeks in subjects with CKD stage 2 and 3. Our principal finding was that low fructose diet reduced blood pressure in those with a “dipping” pattern, as well as fasting serum insulin and CRP levels. There was also a nonstatistical reduction in serum uric acid [10].

## Conclusions

Subjects with CKD and/or metabolic syndrome have higher baseline serum uric acid compared to healthy controls, and both groups show an increase in serum uric acid following a fructose tolerance test. While the absolute values of uric acid are higher during the fructose tolerance test, the change in serum uric acid levels is similar to that observed in healthy controls, and the relative percent increase in serum uric acid is actually higher in healthy controls. Indeed, the change in serum uric acid with fructose did not correlate with the baseline phenotype of the subjects, which included evaluation of metabolic, renal, inflammatory and target organ injury markers. While these studies suggest that the use of the fructose tolerance test is likely to be of limited value in subjects with metabolic syndrome or CKD, they do not dispute the potential importance of long-term fructose intake on metabolic syndrome or kidney disease.

## Additional file

Additional file 1: Table S1. Laboratory data, inflammatory markers and target organ damage indices. Comparison of investigated group: BMI>30 (n=36), CKD (n=14) and control (n=25).

## Abbreviations

CKD: Chronic kidney disease
FTT: Fructose tolerance test
IMT: Intima media thickness
RI: Resistance index
PI: Pulsatility index
AccT: Acceleration time
SBP: Systolic blood pressure
DBP: Diastolic blood pressure
ABPM: Ambulatory blood pressure monitoring
eGFR: estimated glomerular filtration rate
BMI: Body mass index
AMP: Adenosino monophosphoran
URIC ACID: Uric acid, SLE, Systemic lupus erythomatodeus
ATP III: Adult treatment panel III
MCP −1: Monocyte chemoattractant protein 1
TGF-beta: Tissue growth factor –beta
TNF-alpha: Tumor necrosis factor-alpha
ACE-Is: Angiotensin converting enzyme-inhibitors
ARB: Angiotensis receptor bloker
