# Supplementary material for: The fructose tolerance test in patients with chronic kidney disease and metabolic syndrome in comparison to healthy controls
Source: BMC Nephrol. 2015 May 3;16:68. doi: 10.1186/s12882-015-0048-y (PMC4450852; doi:10.1186/s12882-015-0048-y)
Supplement: Additional file 1: Table S1. — Laboratory data, inflammatory markers and target organ damage indices. Comparison of investigated group: BMI>30 (n=36), CKD (n=14) and control (n=25). [file 12882_2015_48_MOESM1_ESM.docx]

**Additional file 1: Table S1. Laboratory data, inflammatory markers and target organ damage indices. Comparison of investigated group: BMI>30 (n=36), CKD (n=14) and control (n=25)**

|  | BMI>30 ^(1)^  N=36 | CKD ^(2)^  N=14 | Control ^(3)^  N=25 | p |
| --- | --- | --- | --- | --- |
|  | Mean ± SD  Median (range) | Mean ± SD  Median (range) | Mean ± SD  Median (range) |  |
| Calcium [mmol/l] | 2,32 ± 0,10 | 2,33 ± 0,09 | 2,25 ± 0,15 | p=ns |
| Phosphorus [mmo/l] | 1,03 ± 0,17 | 1,10 ± 0,27 | 1,34 ± 1,17 | p=ns |
| Serum MCP1 [pg/ml] | 100 (44-275) | 95 (71-348) | 124 (79-237) | 1-2 p=1,0000  1-3 p=0,0116  2-3 p=0,0495 |
| Serum TNF-alpha [pg/mL] | 12,2 (0,4-211) | 9,8 (2,4_24,7) | 13,4 (3,8-646) | p=ns |
| Serum TGF-beta [ng/24h] | 0,089 (0,001-0,948) | 0,105 (0,020-0,300) | 0,064 (0,001-24,48) | p=ns |
| Serum iNOS [U/ml] | 8,11 ± 3,13 | 8,14 ± 1,95 | 6,02 ± 2,71 | 1-2 p=0,9997  1-3 p=0,0272  2-3 p=0,0000 |
| Serum eNOS [pg/ml] | 81,1 (2,1-608) | 76,6 (2,1-389) | 103,2 (3,5-648) | p=ns |
| Serum Endothelin-1 [pg/ml] | 6,6 (0,1-1001) | 4,2 (2,1-15,4) | 6,9 (0,8-1001) | p=ns |
| EPO serum | 27,4 (7,3-408) | 13,2 (8,4-141) | 14,6 (2,9-682) | 1-2 p=0,1438  1-3 p=0,0309  2-3 p=1,0000 |
| Urine Creatinine mg/dl | 96,0 ± 39,6 | 54,6 ± 20,7* | 81,0 ± 38,1 | 1-2 p=0,0100  1-3 p=0,3178  2-3 p=0,1404 |
| Urine Creatinine mg/24h | 1652 ± 654 | 1040 ± 232 | 1382 ± 455 | 1-2 p=0,0096  1-3 p=0,1829  2-3 p=0,2153 |
| Urine uric acid mg/dl | 37,0 ± 15,8 | 17,6 ± 8,1* | 30,9 ± 13,9 | 1-2 p=0,0015  1-3 p=0,2833  2-3 p=0,0382 |
| Urinary Calcium mmol/24h | 4,10 ± 2,19 | 2,39 ± 1,54 | 4,63 ± 1,88 | 1-2 p=0,0658  1-3 p=0,6121  2-3 p=0,0108 |
| Urinary Phosphorus mmol/24h | 34,37 ± 13,91 | 20,08 ± 7,10 | 28,63 ± 11,03 | 1-2 p=0,0067  1-3 p=0,2150  2-3 p=0,1497 |
| NAG U/L | 0,70 (0,35–3,80) | 0,95 (0,35–2,40) | 0,80 (0,25–2,20) | p=ns |
| Cystatin C [ng/ml] | 729 (461-1241) | 1197 (609-3422) | 585 (411-1074) | 1-2 p=0,0010  1-3 p=0,0203  2-3 p=0,0000 |
| IMT [mm] (mean value) | 0,828 ± 0,178 | 0,946 ± 0,136 | 0,746 ± 0,139 | 1-2 p=0,1484  1-3 p=0,1639  2-3 p=0,0056 |
| RI (mean value) | 0,725 ± 0,115 | 0,782 ± 0,189 | 0,683 ± 0,065 | p=ns |
| PI (mean value) | 1,305 ± 0,270 | 1,478 ± 0,639 | 1,233 ± 0,239 | p=ns |
| AccT [ms] (mean value) | 49,7 ± 20,0 | 70,2 ± 35,6 | 54,3 ± 14,0 | p=ns |
| Micro albuminuria[mg/l] | 4,5 (2,0-499) | 20,7 (2,0-1430) | 3,2 (2,1-8,6) | 1-2 p=0,6281  1-3 p=0,1502  2-3 p=0,0200 |
